# Synthaser: a CD-Search enabled Python toolkit for analysing domain architecture of fungal secondary metabolite megasynth(et)ases

**DOI:** 10.1186/s40694-021-00120-9

**Published:** 2021-11-11

**Authors:** Cameron L. M. Gilchrist, Yit-Heng Chooi

**Affiliations:** School of Molecular Sciences, The University of Western Australia, 35 Stirling Hwy, Crawley, 6009 Australia

**Keywords:** Secondary metabolism, Domain architecture, Polyketide synthase, Nonribosomal peptide synthetase, Bioinformatics, software

## Abstract

**Background:**

Fungi are prolific producers of secondary metabolites (SMs), which are bioactive small molecules with important applications in medicine, agriculture and other industries. The backbones of a large proportion of fungal SMs are generated through the action of large, multi-domain megasynth(et)ases such as polyketide synthases (PKSs) and nonribosomal peptide synthetases (NRPSs). The structure of these backbones is determined by the domain architecture of the corresponding megasynth(et)ase, and thus accurate annotation and classification of these architectures is an important step in linking SMs to their biosynthetic origins in the genome.

**Results:**

Here we report synthaser, a Python package leveraging the NCBI’s conserved domain search tool for remote prediction and classification of fungal megasynth(et)ase domain architectures. Synthaser is capable of batch sequence analysis, and produces rich textual output and interactive visualisations which allow for quick assessment of the megasynth(et)ase diversity of a fungal genome. Synthaser uses a hierarchical rule-based classification system, which can be extensively customised by the user through a web application (http://gamcil.github.io/synthaser). We show that synthaser provides more accurate domain architecture predictions than comparable tools which rely on curated profile hidden Markov model (pHMM)-based approaches; the utilisation of the NCBI conserved domain database also allows for significantly greater flexibility compared to pHMM approaches. In addition, we demonstrate how synthaser can be applied to large scale genome mining pipelines through the construction of an *Aspergillus* PKS similarity network.

**Conclusions:**

Synthaser is an easy to use tool that represents a significant upgrade to previous domain architecture analysis tools. It is freely available under a MIT license from PyPI (https://pypi.org/project/synthaser) and GitHub (https://github.com/gamcil/synthaser).

**Supplementary Information:**

The online version contains supplementary material available at 10.1186/s40694-021-00120-9.

## Introduction

Domains are distinct functional and structural units that serve as the evolutionary building blocks of proteins. The majority of proteins found across all kingdoms of life consist of multiple functional domains, with the growth in number of multi-domain protein families far outpacing that of single domain protein families [1, 2]. Multi-domain proteins predominantly arise from the incorporation of new domains at the N or C terminus by genetic events such as gene fusion, fission, duplication and exon shuffling [3–5]. Extensive domain rearrangement over time has led to the diversification of existing proteins, as well as the emergence of novel protein families [6]. Through this process, domains are placed into new molecular contexts where, via their interactions with different combinations of domains, novel functionality can be birthed [7, 8]. In eukaryotes, many multi-domain proteins have evolved from separate single domain proteins catalysing successive steps of biological pathways in prokaryotes, resulting in improved flux and stability of the pathway [9]. Thus, a study of functional domains, as well as a broader analysis of the domain architectures of proteins in which they are found, can be a fruitful approach for identifying novel functionality.

A good case study for the evolution of multi-domain proteins with domain architectures and functions can be found in the biosynthesis of secondary metabolites, which are bioactive small molecules with important applications in medicine, agriculture and other industries [10, 11]. Secondary metabolites are produced by many microorganisms and plants, but are particularly abundant in filamentous fungi [12]. Indeed, recent genomic work has made obvious the extent of the biochemical arsenal encoded by microbial, and particularly fungal, genomes [13, 14]. The biosynthesis of these compounds is orchestrated primarily through the action of large, multi-domain megasynthases; polyketides are synthesized by polyketide synthases (PKSs) and nonribosomal peptides by nonribosomal peptide synthetases (NRPSs). These multi-domain megasynthases generate the chemical backbones of the compound, which are then modified by ‘tailoring’ enzymes, typically encoded by genes neighbouring the megasynthases in the genome, in what are referred to as biosynthetic gene clusters (BGCs). Much of the work done by natural product researchers in past decades has been focused on the hunt for, and characterisation of, novel BGCs, in hopes of finding the next great drug lead.

Megasynthases can be easily identified by the presence of key functional domains. For example, PKSs typically contain a $\beta$-ketoacyl synthase (KS) domain, which is responsible for building the carbon backphone of polyketides through repetitive condensation of short-chain carboxylic acids [15]. There are also deeper levels of classification based on the presence of other functional domains. Iterative PKSs from fungi, for instance, can be classified as highly-, partially- or non-reducing given the absence or presence of domains that catalyse reduction reactions of the polyketide chain. A highly-reducing PKS will synthesize a reduced polyketide chain, whereas a non-reducing PKS would synthesize an unreduced chain. Other domains present within the megasynthase also affect the synthesized product. For example, the PKSs involved in lovastatin biosynthesis, LovF and LovB, both contain methyltransferase domains which add methyl groups during synthesis of the polyketide product [16].

This link between domain architecture and compound has several useful applications. Firstly, given some isolated metabolite, one can narrow down to the synthases likely responsible for its production by looking for a domain architecture that matches the structure of that metabolite. This is one of the first steps when taking a ‘retro-biosynthetic’ approach to identifying a BGC [17]. Indeed, we have used this approach to identify the megasynthases encoding numerous compounds isolated from Australian fungi [18–21]. Inversely, we can predict that synthases with unique domain architectures could potentially produce unique compounds. Previously we outlined genome mining strategies for the discovery of novel secondary metabolites [22]. One strategy is to prioritise BGCs which have partial similarity to known BGCs, in hopes of finding find new analogues of known bioactive compounds; another is to prioritise completely unique BGCs in order to find novel compounds. In either case, analysis of the domain architectures of secondary metabolite megasynthases plays a key role.

There are currently many databases dedicated to the analysis and functional classification of domains. Pfam [23], SMART [24] and PROSITE [25] are three such databases, each storing information about domain family structure and function. There are also larger resources such as the Conserved Domain Database (CDD; [26]) from the National Center for Biotechnology Information (NCBI), or the InterPro database [27], which integrate many of the smaller domain databases. The CDD contains over 50,000 curated entries taken from seven different sources, and the InterPro database stores over 30,000 entries from thirteen different sources, thus making them the most comprehensive tools for domain analysis available today.

However, there are comparatively few resources dedicated to the analysis of domain architecture. The NCBI offers several tools built on the CDD, most notably CD-Search [28], which searches protein sequences against the CDD to identify the functional domains they contain. CD-Search generates graphical outputs which make it easy to visually discern the domain architectures of query sequences. Likewise, the InterPro database can be searched using the InterProScan tool [27], generating similar output. While adequate for analysing individual sequences, these tools quickly become cumbersome when dealing with larger collections of sequences. Additionally, the output generated by these tools, particularly for larger enzymes, can contain hundreds of conserved domain hits, making it difficult to parse. The NCBI also offers other tools linked to CD-Search: the conserved domain architecture retrieval tool (CDART; [29]), which can be used to find proteins with similar domain architectures; and the subfamily protein architecture labeling engine (SPARCLE; [30]), which groups protein sequences with similar domain architectures and links them to curated functional classifications. Sequences are automatically placed into classification groups from SPARCLE after being analysed by CD-Search. More recently, TREND was developed [31], which allows analysis of domain architecture in an evolutionary context. TREND predicts domains by searching either the CDD or Pfam databases, whilst also generating a phylogeny of the input sequences. However, these tools do precisely annotate all domains within query sequences, with smaller domains often being obscured by hits to larger fused multidomain profiles.

Several tools have been developed specifically for the analysis of secondary metabolite megasynthases. One of the original tools built for this purpose was SEARCHPKS [32], which was subsequently rolled into NRPS-PKS [33] and is now available as a part of the structure based sequence analysis of PKS and NRPS (SBSPKS) webserver [34]. It offers prediction of domain architecture for up to 10 sequences at a time via alignment to curated hidden Markov model (HMM) profiles, as well as predictions of substrate specificity and chemistry and comparison to sequences in a database of characterised PKS and NRPS gene clusters. However, it is not available for local installation, nor is it accessable programatically, and at the time of writing, several pieces of functionality are unavailable. The antibiotics and Secondary Metabolite Analysis Shell (antiSMASH) performs rule-based prediction of biosynthetic gene clusters in genomes based on the presence of key seed domains [35]. The domain architectures of megasynthases in predicted BGCs are determined by searching a local database of curated profile HMMs. Occasionally domains are missed in the predicted architecture, particularly smaller domains which typically achieve lower scores during searches (e.g. acyl-carrier protein domains). Additionally, as antiSMASH takes genome sequence as input, it may be unsuitable for analysis of single proteins. The use of internally curated HMM profiles in both SBSPKS and antiSMASH, while greatly improving speed and specificity of predictions, also makes them inflexible to prediction of new domain types.

Here we describe synthaser, a Python based software package leveraging the NCBI’s CD-Search API which can automatically annotate and classify the domain architectures of multi-domain proteins based on a flexible, user-definable ruleset system. Synthaser produces interactive visualisations of proteins grouped by their classification, making proteins with interesting architectures immediately apparent. Below, we extensively detail the synthaser search workflow and other functionality in the package, including modules for downloading search databases and extracting domain sequences, as well as a web application for easily building rule sets. As a proof of concept, we detail the process of building a synthaser ruleset using the web application for the classification of fungal secondary metabolite megasynthases, specifically polyketide synthases and nonribosomal peptide synthases. To evaluate this rule set, we analyse all available PKS and NRPS sequences deposited in the MIBiG repository [36] and compare the domain architectures predicted by synthaser to the corresponding antiSMASH-generated predictions stored in each MIBiG entry. Finally, we build a similarity network of polyketide synthases in publicly available *Aspergillus* genomes, and link it to synthaser domain architecture predictions to demonstrate how synthaser can be used to quickly identify interesting sequence groups for further investigation. We show synthaser to be a useful addition to the genome mining toolbox, particularly within the context of natural products research; however, given the programmable nature of synthaser, we can foresee much broader applications of the software.

## Materials and methods

### Software implementation and availability

Synthaser is implemented in Python 3, and only requires the requests library to perform remote searches. Synthaser is open source and is made freely available on GitHub (https://github.com/gamcil/synthaser) and PyPI (https://pypi.org/project/synthaser) under a MIT license. To perform local searches, synthaser requires that both Reverse Position Specific BLAST (RPS-BLAST) as well as rpsbproc, the command line utility that formats local RPS-BLAST results to resemble those returned by the CD-Search web service, are installed and accessable on the system [37].

### The synthaser search workflow

**Fig. 1:**
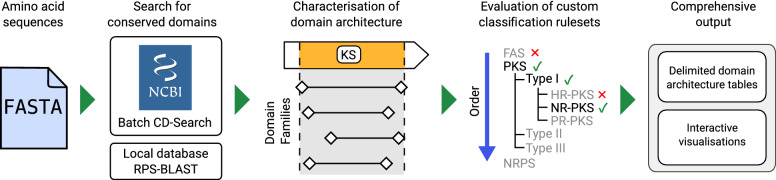
Outline of the synthaser workflow

The synthaser search workflow is detailed in Fig. 1. Briefly, query sequences are read from FASTA files and sent to the NCBI’s CD-Search API to search for functional domains (or RPS-BLAST in local searches). Domain architectures of query sequences are annotated based on an analysis of overlapping domains. Sequences are classified according to a hierarchy of rules encoded in a programmable rule file. Finally, synthaser produces comprehensive text and visual outputs. These steps are described in more detail below.

#### Accepted input

Synthaser accepts files in FASTA format, as well as collections of valid NCBI sequence identifiers specified either in newline-separated text files or directly passed to the command line interface. Query sequences are parsed directly from FASTA files using BioPython [38], whereas sequences corresponding to NCBI identifiers are retrieved using the Entrez API [39]. Additionally, synthaser provides a module, genbank, which allows users to extract all PKS and NRPS sequences from GenBank format files generated by antiSMASH (version 5.0 and above) to a FASTA file ready for synthaser analysis.

#### Remote searches via NCBI Batch CD-Search API

In remote searches, query sequences are uploaded to the BATCH CD-Search API [40]. Every search is assigned a unique CD-Search identifier (CDSID) that is saved and reported in the output. Each CDSID remains valid for 36  h, and can be used to directly re-start a synthaser run at any point during this period. The CDSID is polled against the API continuously until the search has completed and results can be retrieved.

#### Local searches using RPS-BLAST and rpsbproc

The underlying search for any remote CD-Search run is performed using Reverse Position Specific BLAST (RPS-BLAST), a variant of Position-Specific Iterated BLAST (PSI-BLAST), which searches protein sequences against a database of domain profiles [41]. By default, RPS-BLAST output resembles the output of other BLAST variants. The NCBI offers another tool, rpsbproc, which processes RPS-BLAST results to resemble those returned by the CD-Search web server [30]. Synthaser provides a local search mode which wraps RPS-BLAST and rpsbproc, enabling searches against local profile databases. Here, input sequences are searched against a local profile database using RPS-BLAST, and search results are post-processed by rpsbproc such that they can be analysed like remote CD-Search results. The domain family profile databases used in CD-Search are available as pre-formatted RPS-BLAST databases from the NCBI FTP server, which can be retrieved using the getseq module.

#### The central synthaser rule file

Once CD-Search results have been retrieved, synthaser undergoes two phases: identification of domains in query sequences, and the classification of query sequences. Underlying these phases is a central rule file in JSON format which specifies (i) conserved domains that synthaser should attempt to identify in query sequences, (ii) rules for assigning classifications to sequences based on identified domains, and (iii) a hierarchy that determines the order of rule evaluation. The full schema of the rule file is detailed in Additional file 1: Fig. S1.

#### Identification of functional domain ‘islands’

**Fig. 2:**
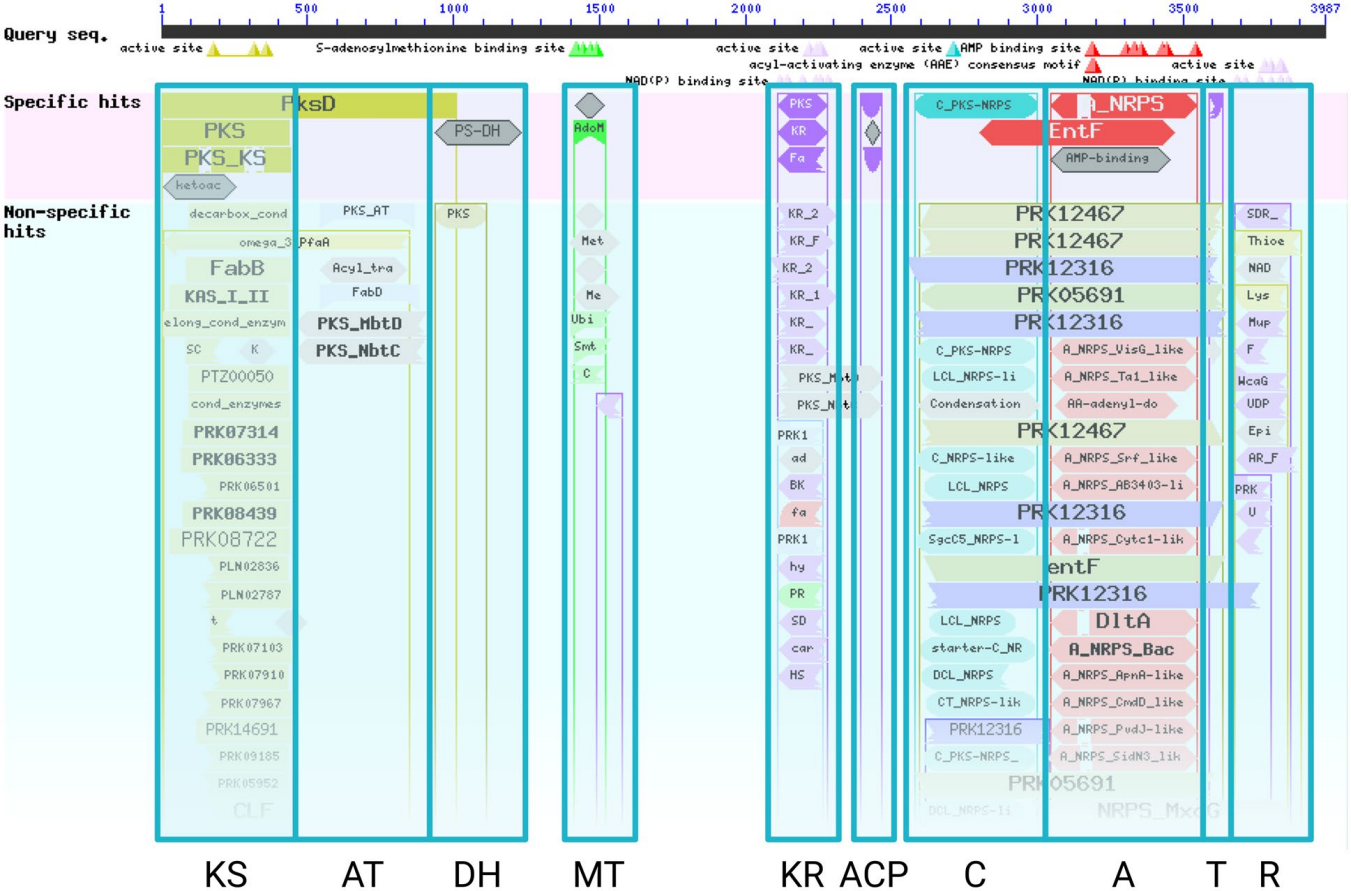
Naturally segragating ‘islands’ of functional domains found in CD-Search analysis of BuaA, the hybrid PKS-NRPS involved in the biosynthesis of the burnettramic acids [19]. The islands correspond to the domain architecture KS-AT-DH-MT-KR-ACP-C-A-T-R

Domain hits in CD-Search results naturally segregate into distinct ‘islands’ of overlapping related domain families (Fig. 2). Synthaser attempts to characterise the domain architecture of query sequences by programmatically identifying these islands. This is done by defining sets of conserved domain families that correspond to broader functional classes (as specified in the rule file). For example, the KS island in Fig. 2 consists of a variety of individual conserved domain families (e.g. PKS, PKS_KS, KAS_I_II). These values are used at several stages in the synthaser workflow.

During the domain identification phase, synthaser discards domain hits not specified by any broader domain class and filters remaining hits for quality. Every domain family in the CDD has an underlying position-specific scoring matrix (PSSM) describing the amino acid makeup of the family, as well as a threshold bit-score value used to determine if a given hit is a specific (i.e. high confidence) hit. By default, synthaser will discard hits that do not meet the thresholds for PSSM length (30% of the family PSSM), or bitscore (30% of specific-hit bitscore); users can freely adjust these parameters. After filtering, synthaser identifies groups of overlapping domain hits, choosing a representative hit based on maximum length, maximum bitscore or minimum e-value.

Occasionally, a single-domain can be reported as short, discontiguous, low-scoring hits. To resolve such cases, synthaser explicitly checks for adjacent, truncated domain hits of the same or equivalent types. Synthaser uses two threshold parameters to determine if merging should occur: (i) the length of each hit as a proportion of their corresponding PSSM lengths, termed coverage, and (ii) the bitscore of each hit as a proportion of the specific-hit PSSM. Two hits are merged if both occur within the space of a single PSSM length ($\pm 10\%$), their combined bitscore is above the threshold bitscore, and the combined lengths are above a given query coverage threshold.

Finally, synthaser reports the domain architecture of each query sequence.

#### Functional classification based on domain architecture

Once the domain architectures of query sequences have been characterised, synthaser has the option to evaluate the classification rules defined in the rule file. This allows for the division of multidomain proteins into subgroups based on the absence or presence of specific domains, which can provide additional insights into the functional differences between them. Each rule must contain (i) a name, (ii) a list of domain types, and (iii) a logical expression used to evaluate the rule, hereafter termed an evaluator. The rule name is transferred to the sequence upon successful evaluation; each sequence has a classification array which can contain any number of rule names (i.e. multiple rules satisfied in hierarchy). The list of domain types contains domains which are referenced by, though not necessarily required for satisfaction of, the rule. The evaluator is a logical expression which determines if a rule is satisfied by a collection of domains. It is comprised of a series of numbers referring to the indices of each domain in the list of domain types and logical operators that connect them. When a rule is evaluated on a collection of domains, synthaser checks that domains referred to in the evaluator are found, substituting the corresponding numerical index in the evaluator with the result (True if the domain type is found, otherwise False). The final expression is then evaluated to determine if the rule has been satisfied or not.

Continuing the example shown in Fig. 2, we may wish to create a PKS-NRPS rule which requires domain types KS and A. In this rule, the domains list may resemble 1:

1 $$\begin{aligned} {[}KS,\ A] \end{aligned}$$

As both domain types are required for the rule, the evaluator would then resemble 2:

2 $$\begin{aligned} 0\ and\ 1 \end{aligned}$$

Here, 0 refers to the KS domain and 1 refers to the A domain. If analysing a PKS containing a KS domain but not an A domain, the evaluator after substitution would resemble 3:

3 $$\begin{aligned} True\ and\ False \end{aligned}$$

As 3 evaluates to False, the rule is not satisfied. However, classifying the sequence in Fig. 2 would yield the expression 4:

4 $$\begin{aligned} True\ and\ True \end{aligned}$$

Thus successfully classifying the sequence as a PKS-NRPS.

Rules can additionally include specific domain orders, domain type filters and renaming rules. A rule with a domain order will not be satisfied unless the required domains occur in the sequence in the specified order. This is helpful in cases such as hybrid PKS-NRPSs and NRPS-PKSs, where the correct classification is dependent upon the order of the PKS and NRPS modules within the sequence. Domain type filters indicate that a rule only accepts a domain in a sequence if the representative hit is of a specific domain family. This allows for differentiation between specific families that fall under the same broader classes (e.g. KS domains from FAS and PKS). Renaming rules allow domain types to be renamed in synthaser output. This is useful in cases where functionally equivalent domains have different nomenclature based on context. For example, acyl carrier proteins (ACP) of PKSs and peptidyl carrier proteins (PCP) of NRPSs are closely related and typically hit the same domain families in a CD-Search, but convention dictates they are denoted by ACP in PKSs and T (thiolation) or PCP in NRPSs. Renaming rules can optionally include *before* or *after* domains, which specify that the renaming target should only be renamed if it is found before or after certain domains. For instance, an ACP in a hybrid PKS-NRPS should only be renamed to T within the NRPS module, which can be accounted for in the rule by adding key NRPS domains (e.g. A or C) as *after* domains.

**Fig. 3:**
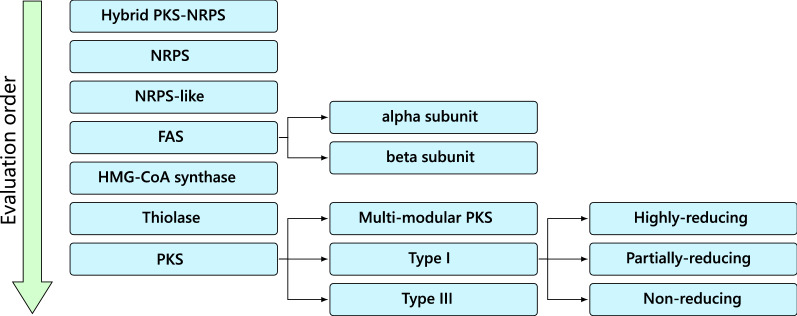
Evaluation hierarchy of sequence classification rules in fungal secondary metabolite megasynthases. Rules in this hierarchy are evaluated top-to-bottom; if a rule evaluates unsuccessfully, the next rule in the hierarchy is evaluated. If a rule with children (relationship depicted by arrows) is successfully evaluated, the child rules will be evaluated. Upon successful evaluation, the full classification array, including the parent and all child rules, is saved on the sequence

The final element of the classification rule system is the hierarchy. This takes the form of a tree structure, with each node in the tree containing the name of a classification rule as well as a list of any child rules (Fig. 3). Synthaser uses this tree to determine the order of evaluation during sequence classification. If a rule is not satisfied, synthaser will proceed to the next rule of the same depth within the tree; if it is satisfied, synthaser will recurse into the children of that rule, and so on. During this stage of the synthaser workflow, the rule hierarchy is evaluated on each sequence, which is then assigned a classification array containing the names of all rules which were succesfully evaluated. For instance, the default rule file would assign a highly-reducing PKS the array: [*PKS, Type I PKS, Highly-reducing PKS.*] After sequences have been classified, domain architectures of each query sequence is reported, and an interactive visualisation is generated.

The generic nature of this rule system means that although synthaser was written primarily to analyse secondary metabolite megasynthases, it can be readily repurposed for the analysis of any multi-domain protein family.

### Building rule files using the synthaser rule generator web application

**Fig. 4:**
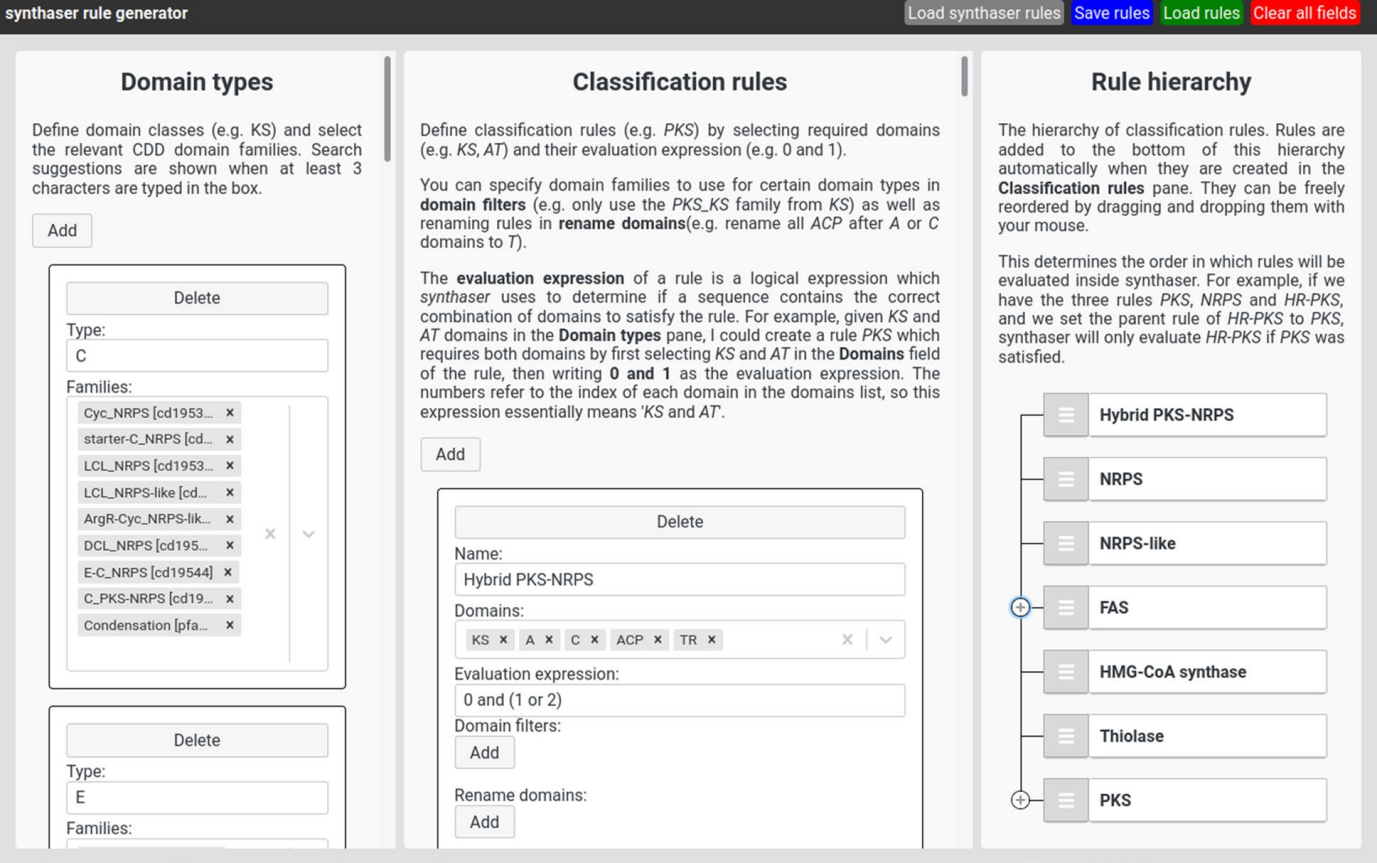
Web application for designing and editing synthaser rule files. Rule files are built in three stages: domain classes are defined in the ‘Domain types’ pane; sequence classification rules are built in the ‘Classification rules’ pane; and the hierarchy of rule evaluation is determined by re-arranging the rules in the ‘Rule hierarchy’ pane. The web application is hosted at https://gamcil.github.io/synthaser

Given how cumbersome it is to manually assemble the synthaser rule file, we provide a web application which can generate rule files in three easy steps (Fig. 4).

In the leftmost pane, the collection of domain types are built by specifying names (e.g. *KS*) and domain families (*PKS* and *PKS_KS*). The *Families* selection box is linked to a file containing information about every family in the CDD, so families can be found simply by searching their names or accessions in the box.

Once domain types have been created, classification rules can be built in the middle pane. Each rule requires a name, a list of domain types it requires and the evaluator. The *Domains* box allows for selection of the domain types created in the *Domain types* pane. Rule names and evaluators can be added simply by writing in the relevant field. Domain type filters can be added inside the *Domain filters* section of each rule. Within each filter, the domain type can be specified in the *Domain name* selection field, and the domain families in the *Domain types* selection field. Renaming rules can be added in the *Rename domains* section. Within each renaming rule, the renaming target domain can be selected in the *From* field, any after domains in the *After* selection field, and the new name in the *To* input field.

Finally, the classification rule hierarchy can be established in the rightmost pane. When a rule is added or updated in the *Classification rules* pane, it is automatically added to, or updated in, the rule hierarchy. Each rule can be dragged and dropped anywhere within the hierarchy, and can be easily nested to form parent-child rule relationships.

The default ruleset bundled with synthaser can be loaded by clicking the *Load default rules* button (Fig. 4d), so users can quickly understand how the rule generator system works. After building the ruleset in the web application, the JSON rule file can be generated by clicking the *Save rules* button; this can be easily loaded back into the application using the *Load rules* button, enabling easy updates to pre-existing rule files. This file can be passed to the synthaser search module using the -uf / –rule_file argument, where it will be loaded in place of the default ruleset.

The web application is implemented using the React framework and is hosted on GitHub pages (https://gamcil.github.io/synthaser).

### Reporting and visualisation

Synthaser provides comprehensive textual and visual outputs. By default, textual results are generated and printed to the command line. In this output, query sequences are listed alongside their predicted domain architecture, grouped by their classification groups and in descending order of sequence length. Tabular, long form versions of this output can also be easily generated, allowing further analysis in spreadsheet software.

**Fig. 5:**
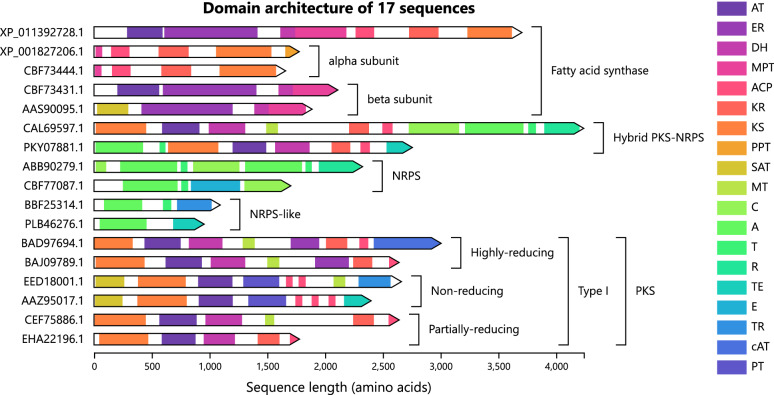
Synthaser visualisation of a subset of the analysed synthase sequences sourced from the MIBiG database covering a spectrum of classification groups

Synthaser can also generate an interactive visualisation by using the –plot argument (Fig. 5). Each query sequence is drawn to scale, grouped by classification and shown in descending sequence length order. Box annotations are drawn for each classification group; nested classifications are shown in reverse order (deepest classifications in the hierarchy shown first), making broader classification groups immediately obvious. Each sequence contains a collection of domains; all unique domain types within the visualisation are assigned a colour, which is used to colour both the domain element within the sequence, as well as the corresponding entry within the legend. Hovering over a domain will produce a tooltip which contains a summary of information about the domain hit, including the specific CDD family, its superfamily (if applicable), the domain class as determined by the rule file, its position within the query sequence, and the E-value and bitscore values from the CD-Search results. The sequence of the domain hit within the query can be copied to the system clipboard directly by clicking a button within the tooltip; the entire query sequence can also be copied in this way. FASTA files containing all sequences of a certain domain type can easily be generated within the *Download domain sequences* section of the settings panel, by first selecting the desired domain type and then clicking the download button.

The synthaser visualisation has various settings available to tweak its appearance. The shape, size and positioning of each synthase, as well as the vertical spacing between synthases, can be manipulated; maximum sequence length in pixels can be adjusted to control the width of the plot; and the font size of the various text elements within the plot, as well as text and box elements within the legend, can be changed. Once the user is satisfied with the appearance of the plot, a scalable vector graphics (SVG) image file can be generated by clicking the *Save SVG* button at the top of the settings panel. This file can be directly imported into vector image software for further manual editing.

Synthaser can also generate a static HTML document containing all data and code required to display the visualisation when a file name is provided to the –plot argument. This enables synthaser results to easily be shared between individual computers.

The visualisation is implemented using the D3 JavaScript library [42], and is available as a standalone reusable chart library under the MIT license (https://www.github.com/gamcil/synthaser.js).

### Analysis of characterised synth(et)ases

Characterised PKS clusters of fungal origin were obtained from MIBiG 2.0 [43]. The corresponding GenBank and JSON files (obtained from the full database dumps for each format) were then parsed for PKS and NRPS sequences, as well as their corresponding domain architectures as annotated on MIBiG, using a Python script (available from https://github.com/gamcil/synthaser_scripts) and then added to the dataset. Sequences were then analysed using synthaser with default settings (maximum E-value 1.0, domain family PSSM length threshold percentage 40%, domain family bitscore threshold percentage 40%, domain family coverage 60%, tolerance 10%), and compared to the antiSMASH domain architecture predictions shown by MIBiG. Sequences were compared for overall domain architecture matches between synthaser and antiSMASH predictions stored in MIBiG. Discrepencies were sorted into groups based on the specific type of mismatch between each prediction: domains identified in synthaser but not in MIBiG; equivalent domains found but mis-named in MIBiG; and mis-named in synthaser predictions. Count data was analysed and visualised using R.

### Building a network of *Aspergillus* polyketide synthases

**Fig. 6:**
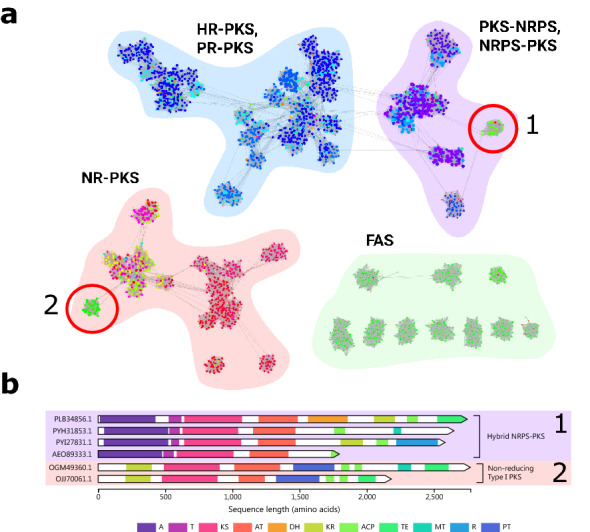
Ketosynthase (KS) domain similarity network of polyketide and fatty acid synthases in *Aspergillus* genomes (**a**) and synthaser domain architecture analysis of representative sequences taken from two groups of interest identified in the network (**b**). Colours of nodes correspond to the full domain architectures of each synthase

Sequences containing ketosynthase (KS) domains were identified by querying the NCBI Protein database for entries linked to the ‘cond_en-zymes’ (CDD UID: 238201) superfamily using Entrez Direct [44]. The results were filtered to only include GenBank sequences from *Aspergillus* species. All remaining sequences were retrieved, and were analysed for PKS domains with the built-in ruleset using default settings with the command ‘synthaser search -qf sequences.fa’. Regions corresponding to KS domains in the identified sequences were extracted using the extract module in synthaser. The extracted sequences were formatted as a DIAMOND database and aligned against themselves using DIAMOND 0.9.17 with the ‘more-sensitive’ flag [45]. An edge table was generated by summing the bitscores of individual high-scoring segment pairs (HSPs) of each unique query and target sequence pair using a custom Python script. This table was imported into Cytoscape 3.7.2, and a Prefuse Force Directed Layout using the summed bitscores of domain-domain BLAST alignments was applied to the generated network. Representative domain architectures of each cluster were identified by mapping synthaser results to the extracted domains in the Cytoscape network. A discrete colour scheme based on an alphabetical ordering of all domain architectures in the network was generated in CytoScape and applied to the network (resulting colours shown in Fig. 6). A full explanation of the creation of the network, as well as custom Python scripts and intermediate analysis files, has been provided on a GitHub repository (https://github.com/gamcil/synthaser_scripts).

## Results

### A classification framework for fungal megasynthases

Two of the major classes of natural products are polyketides and nonribosomal peptides, synthesized by polyketide synthases (PKS) and nonribosomal peptide synthetases (NRPS), respectively [10]. There is significant interest in the genome mining of new polyketide and nonribosomal pathways for their potential in making new drugs [11]. These megasynthases are large enzymes consisting of multiple functional domains, each responsible for a different step in the biosynthesis of the products backbone.

PKSs, similar to fatty acid synthases (FAS), build the carbon backbone of polyketides through repetitive condensation of short-chain carboxylic acids, catalyzed by a $\beta$-ketoacyl synthase (KS) domain [15]. KS domains belong to a broader family of condensing enzymes, which includes enzymes catalysing decarboxylating and non-decarboxylating reactions [46]. The decarboxylating enzymes are further broken into the ‘initiation’ enzymes, which include chalcone synthases (CHS) of Type III PKSs and hydroxymethylglutaryl (HMG)-CoA synthases, and ‘elongation’ enzymes, which include $\beta$-ketoacyl-ACP synthases (type I and II) of FAS and KS domains of PKS. The non-decarboxylating enzyme group is comprised of biosynthetic and degradative thiolases.

PKSs are generally classified as types I, II or III (Table 1), though only types I and III are found in fungi. A minimal PKS consists of the KS, as well as acyltransferase (AT) and acyl-carrier (ACP) domains required for chain extension. While bacterial type I PKS are typically modular, with each chain extension step encoded by a distinct module, fungal PKS are typically iterative, with a single module being used repeatedly; though examples of modular PKS have been identified in fungi [10, 47]. Iterative type I PKS are further classified as highly-reducing (HR), partially-reducing (PR) or nonreducing (NR) based on the presence of reductive $\beta$-keto processing domains. HR-PKSs typically produce aliphatic or alicyclic compounds, and will contain enoylreductase (ER), ketoreductase (KR) and dehydratase (DH) domains, which catalyze reduction reactions on the $\beta$-keto group during each chain extension step. Notable HR-PKSs include LovF, the lovastatin diketide synthase involved in the biosynthesis of lovastatin in *Aspergillus terreus* [48] and the prosolanapyrone synthase (PSS) involved in biosynthesis of solanapyrones in *Alternaria solani* [49]. PR-PKS contain at least one, but not all, of these reductive domains [50]. For instance, the well known 6-methylsalicylic acid synthase (6-MSAS) from *Penicillium patulum* [51] and mellein synthase responsible for production of (R)-mellein in *Parastaganospora nodorum* [52] possess DH and KR domains but no ER domains. NR-PKSs have no reductive domains, and typically contain a starter unit:ACP transacylase (SAT), a product template (PT) and a releasing domain (thioesterase (TE) or thioreductase (R) domain) [15]. NR-PKSs almost always produce aromatic compounds, where cyclisation is mediated by the PT domain. For example, *pksA*, involved in the biosynthesis of aflatoxin in *Aspergillus parasiticus* is an NR-PKS [53]. Such classifications are useful as it gives an indication as to the type of compound that may be produced by the PKS.

**Table 1:** Classification scheme of polyketide synthases (PKS) and nonribosomal peptide synthetases (NRPS)

Classification			Key domains
Level 1	Level 2	Level 3	
Hybrid PKS-NRPS			KS, A or C
NRPS			A, T, C
NRPS-like			A
Fatty acid synthase (FAS)			$\beta$-ketoacyl-ACP synthase
	Alpha subunit		ACP, KR, KS
	Beta subunit		SAT, ER, DH
HMG-CoA synthase			HMG-CoA synthase
Thiolase			Thiolase
Polyketide synthase (PKS)			KS
	Multi-modular		Multiple KS
	Type I		KS, AT
		Highly-reducing	ER, KR, DH
		Non-reducing	SAT, PT
		Partially-reducing	ER, KR or DH
	Type III		CHS

Type III PKS are distinguished by their lack of ACP domain and are related to the chalcone and stilbene synthases found in plants [54]. They are observed mostly in bacteria, though several have been characterised in fungi and have been shown to produce $\upalpha$-pyrones, resorcylic acids and resorcinols [55, 56].

NRPSs typically consist of multiple modules, each possessing a binding specificity to a specific amino acid, which can be proteinogenic or non-proteinogenic. A nonribosomal peptide is synthesized through the formation of peptide bonds between amino acids attached to adjacent modules [10]. A minimal NRPS consists of adenylation (A), peptidyl carrier protein (PCP)/thiolation (T), condensation (C) and thioesterase (TE) domains, and can be modular or iterative.

Finally, it is possible to have hybrid enzymes that contain both PKS and NRPS modules (denoted PKS-NRPS, or NRPS-PKS depending on module order) which in turn produce polyketide-peptide metabolites, or pathways of a mixture of PKS types [54]. Notable examples include the PKS-NRPSs involved in the biosynthesis of the burnettramic acids in *Aspergillus burnettii* [19], the cytochalasins in *Aspergillus clavatus* [57], and phomacins in *Parastaganospora nodorum* [58].

### Building a synthaser ruleset

Multidomain protein families can often be divided into subgroups based on the absence or presence of certain domains, which can facilitate further functional predictions. Likewise, fungal type I PKSs have been subdivided into HR, PR and NR-PKS based on the absence or presence of reductive $\beta$-keto processing domains. This information provides insights into the nature of the polyketide products encoded by the PKS genes; for instance, NR-PKS are most likely to make aromatic compounds, while HR-PKS can make alicyclic or aliphatic compounds. As a proof of concept for the synthaser workflow, we designed a rule file for the classification of fungal megasynthases, namely PKS-NRPS, FAS, PKS and NRPS. Using the rule generator web application, we built the rule file according to the classification scheme shown in Table 1. A synthaser ruleset is comprised of three elements: the domain classes that we wish to identify, rules to classify sequences based on the domain classes that are identified, and a hierarchy which determines the order in which rules are evaluated. The domain classes, as well as the domain families that comprise them and scoring information is shown in Table 2. In sum, 58 domain families were placed into 16 different domain classes, covering domains frequently observed in fungal secondary metabolite megasynthases. This included classes for adenylation (A), acyl-carrier protein (ACP), ACP synthase (ACPS), acyltransferase (AT), condensation (C), dehydrogenase (DH), epimerization (E), enoylreductase (ER), ketoreductase (KR), beta-ketoacyl synthase (KS), methyltransferase (MT), product template (PT), starter unit:acyl carrier protein transacylase (SAT), thioesterase (TE), thioester reductase (TR) and carnityl acyltransferase (cAT). Domain families for each class were manually chosen by performing online CD-Search searches with characterised megasynthase sequences and analysing which domain families appear in each domain ‘island’ observed in the visual output (see Fig. 2).

**Table 2:** Domain classes and domain families defined in the default synthaser rule file

Domain class	Domain family				
	Accession	Name	PSSM ID	PSSM Length	Threshold bitscore
A	cd05930	A_NRPS	341253	444	356.838
	pfam00501	AMP-binding	366135	361	184.727
ACP	smart00823	PKS_PP	214834	86	33.3777
	CHL00124	acpP	177047	82	85.8428
	pfam14573	PP-binding_2	373139	96	112.899
	pfam00550	PP-binding	376348	67	29.814
ACPS	COG0736	AcpS	223807	127	88.0777
	PRK00070	acpS	234610	126	87.108
AT	smart00827	PKS_AT	214838	298	201.477
C	cd19535	Cyc_NRPS	380458	423	348.324
	cd19533	starter-C_NRPS	380456	419	482.254
	cd19538	LCL_NRPS	380461	432	638.54
	cd19531	LCL_NRPS-like	380454	427	287.329
	cd20480	ArgR-Cyc_NRPS-like	380470	406	714.278
	cd19543	DCL_NRPS	380465	423	420.456
	cd19544	E-C_NRPS	380466	413	476.159
	cd19532	C_PKS-NRPS	380455	421	402.605
	pfam00668	Condensation	334202	455	345.862
DH	pfam14765	PS-DH	379688	291	132.479
	smart00826	PKS_DH	214837	167	76.8814
E	cd19534	E_NRPS	380457	428	363.495
ER	COG4981	COG4981	227314	717	1062.53
	smart00829	PKS_ER	214840	287	250.768
	cd05195	enoyl_red	176179	293	129.997
	cd08270	MDR4	176231	305	268.471
	cd05282	ETR_like	176645	323	224.079
KR	smart00822	PKS_KR	214833	180	83.3005
	cd08950	KR_fFAS_SDR_c_like	187653	259	368.441
KS	smart00825	PKS_KS	214836	298	241.079
	cd00833	PKS	238429	421	167.35
	cd00829	SCP-x_thiolase	238425	375	147.795
	TIGR01833	HMG-CoA-S_euk	273826	457	790.507
	cd00751	thiolase	238383	386	222.354
	PLN02287	PLN02287	215161	452	632.955
	PRK07314	PRK07314	235987	411	601.393
	TIGR03150	fabF	274452	407	525.512
	COG0304	FabB	223381	412	152.8
	cd00832	CLF	238428	399	503.431
	cd00834	KAS_I_II	238430	406	285.201
	cd00831	CHS_like	238427	361	242.515
	cd00830	KAS_III	238426	320	212.4
MT	pfam08241	Methyltransf_11	369777	93	53.4302
	pfam08242	Methyltransf_12	369778	96	37.7331
	pfam13489	Methyltransf_23	372616	162	59.7422
	pfam13649	Methyltransf_25	379312	96	36.008
	pfam13847	Methyltransf_31	316372	150	67.449
	cd02440	AdoMet_MTases	100107	107	29.3203
	smart00828	PKS_MT	214839	224	133.695
PT	TIGR04532	PT_fungal_PKS	275325	324	202.466
SAT	pfam16073	SAT	374347	239	110.757
TE	smart00824	PKS_TE	214835	212	155.846
	pfam00975	Thioesterase	366397	223	155.591
	COG0657	Aes	223730	312	59.5636
	pfam00561	Abhydrolase_1	366166	245	93.3378
TR	TIGR01746	Thioester-redct	273787	367	305.493
	cd05235	SDR_e1	187546	290	229.845
cAT	pfam00755	Carn_acyltransf	376382	577	255.551

**Table 3:** Overview of rules for classification of fungal secondary metabolite megasynthases used in synthaser

			Domain rules		Rename rules			
Rule name	Domains	Evaluator	Class	Families	From	To	Before	After
Hybrid PKS-NRPS	KS, A, C, ACP	0 and (1 or 2)			ACP	T		A, C
					T	ACP	A, C	KS
Thiolase	KS	0	KS	cd00829, cd00751, PLN02287				
HMG-CoA synthase	KS	0	KS	TIGR01833				
beta subunit	AT, ER, DH	0 and 1 and 2	ER	COG4981				
alpha subunit	AT, KR, ACPS	0 and 1 and 2	KR	cd08950				
FAS	KS	0	KS	COG0304, cd00834, cd00830, TIGR03150				
NRPS-like	A, C, ACP	0 or 1			ACP	T		
NRPS	A, ACP, C	0 and 2			ACP	T		
Type III	KS	0	KS	cd00831				
Non-reducing PKS	SAT, PT	0 and 1						
Partially-reducing PKS	DH, ER, KR	0 or 1 or 2	KR	smart00822, cl00100				
			ER	smart00829, cd05195				
			DH	smart00826				
Highly-reducing PKS	DH, ER, KR	0 and 1 and 2	KR	smart00822, cl00100				
			ER	smart00829, cd05195				
Type I PKS	AT	0	AT	smart00827, cl08282				
Multi-modular PKS	KS, KS	0 and 1						
PKS	KS	0	KS	smart00825, cd00833, cd00831				

Overview of rules for classification of fungal secondary metabolite megasynthases used in synthaser. Each rule is comprised of a name, a set of domain classes, an evaluator, domain filters which specify valid domain families for a given domain class, and rename rules, which specify domain classes which should be renamed in certain contexts

Once domain classes had been established, functional classification rules could be created. Following the framework outlined in Table 1, we generated a collection of rules covering each unique megasynthase classification (Table 3). In total, 15 rules were created, covering the spectrum of fungal PKS, fatty acid synthase (FAS) and NRPS sequences. These consist of 7 top-level rules, including those for Hybrid PKS-NRPS, Thiolases, HMG-CoA synthases, FAS, NRPS, NRPS-like and PKS sequences.

Within these top-level rules, there are further child rules. For example, FAS sequences are further classified as alpha or beta subunit. Similarly, PKS sequences can be classified as multi-modular (containing multiple KS domains), Type I or Type III; Type I sequences can be further classified into highly-, partially- or non-reducing PKS.

Finally, a rule evaluation hierarchy was created (Fig. 3). Synthaser evaluates from the first listed rule to the last, recursing into child rules if successful. This makes it simple to define hierarchies with any number of levels where rules incrementally build on other rules to assign more specific classifications.

The final rule file is distributed alongside the source code and is freely available from the GitHub repository.

### Analysis of megasynthases in characterised biosynthetic gene clusters

In order to verify the accuracy of our fungal secondary metabolite megasynthase rule set, we decided to test it against previously characterised megasynthases deposited in the MIBiG database [36]. BGCs of fungal origin were retrieved from the MIBiG database, and 284 sequences covering the spectrum of fungal megasynthase classifications were extracted (Additional file 2: Table S1). Domain architectures of a subset of these sequences is shown in Fig. 5. This collection consisted of 137 PKS, 61 NRPS, 31 NRPS-like, 24 FAS and 31 hybrid PKS-NRPS sequences (as determined by synthaser classification). Of the 137 PKS sequences, 48 were further classified as highly-reducing, 70 as non-reducing and 16 as partially-reducing. Similarly, of the 24 FAS sequences, 23 could be further classified into separate alpha (12 sequences) and beta-subunit (11 sequences) encoding genes, typical of the Ascomycetes, with the remaining sequence, *fas2* from the ustilagic acid BGC in *Ustilago maydis*, being a complete single-chain FAS [59], common to the Basidiomycetes and mycobacteria [60].

All sequences were correctly classified. Domain architectures predicted by synthaser either matched completely or identified more domains than the antiSMASH predictions reported on MIBiG; different naming schemes for equivalent domains, for example ACP, PCP and T domains, were not considered mismatches in this comparison. In total, 182 (64.08%) antiSMASH-generated domain architecture predictions exactly matched those from synthaser. Of the remaining 102 predictions (35.92%), 101 were mismatched due to domains being present in the synthaser predictions but not in the MIBiG records; synthaser reported one extra domain in 75 (26.41%) cases, two in 38 (13.38%) cases, and three in 24 (8.45%) cases. The most frequent of these extra domains were ACP/T domains, in PKS and NRPS, respectively, which were absent in 32 (11.27%) of the antiSMASH-generated predictions, as well as TE domains (16, 5.63%), KR domains (14, 4.93%) and SAT domains (14, 4.93%).

Notably, synthaser architecture predictions for the sordarin HR-PKS from *Sordaria araneosa* [61], and the AF-toxin HR-PKS from *Alternaria alternata* [62], both contain carnitine acyltransferase (cAT) domains, which are not present in the antiSMASH-generated domain architecture predictions. The cAT domain was recently shown to be capable of esterification of polyketide products in *Trichoderma virens* [63]. While the Pfam database contains a profile HMM corresponding to the cAT domain (accession: PF00755), the NRPS/PKS analysis module in antiSMASH currently does not. The extensibility of the synthaser rule system allows for new domains to be easily added, provided an entry is available within the CDD.

Another notable case study is the starter unit:acyl carrier protein (ACP) transacylase (SAT) domain, a characteristic feature of NR-PKSs that is sometimes missed in both the synthaser and antiSMASH-generated domain architecture predictions. For instance, the PKS involved in the biosynthesis of the meroterpenoid paraherquonin in *Penicillium brasilianum* [64], *prhL*, though correctly classified as non-reducing, lacks an SAT domain in both the synthaser and antiSMASH predictions. This is also observed in *trt4*, involved in the biosynthesis of another meroterpenoid compound, terretonin, in *Aspergillus terreus* [65]. As these sequences contain PT domains, they are still correctly classified as NR-PKSs by synthaser as the NR-PKS classification rule requires only one of the key domains (either SAT or PT) to be present for it to be satisfied. On the other hand, predictions for the NR-PKSs of the related andrastin A and novofumigatonin biosynthetic pathways in *Penicillium roqueforti* [66] and *Aspergillus novofumigatus* [67], respectively, do contain SAT domains. Closer inspection of the sequences with missing SAT domains showed annotation gaps in N-terminal regions, indicating that there were likely SAT domains that were missed (Additional file 1: Figure S2). Sequence alignment of the N-terminal regions of NR-PKSs involved in fungal meroterpenoid biosynthesis, annotated both with and without SAT domains, revealed the presence of the characteristic SAT domain active site GXCXG motif [68] in all sequences, confirming that the underlying CD-Search predictions did in fact miss the SAT annotations (Additional file 1: Figure S3). In cases where a domain is missed due to low quality, this problem can be alleviated by simply raising the E-value cutoff used during a synthaser search; in other cases, missing domains may persist due to other reasons (e.g. structural variation, poor domain curation). That the quality of synthaser predictions and classifications is reliant upon the quality of the underlying search databases is a limitation of the tool. However, as the quality of domain profile HMMs in the CDD increases, so too will the power of synthaser to predict and classify domain architectures.

### Network analysis of PKS domain architectures in *Aspergilli* reveals interesting variation

Synthaser can rapidly extract PKS and NRPS genes and generate domain annotations from genome files, making it extremely useful in providing an overview of the diversity of PKS/NRPS domain architectures encoded in an organism. For instance, we recently used synthaser to analyse synthases found in the genome of *A. burnettii*, which facilitated the linkage of expressed metabolites to their corresponding synthases [69]. However, we hypothesized that synthaser could also be incorporated into larger scale genome mining pipelines to guide the discovery of novel metabolites.

To test this hypothesis, we constructed a similarity network of ketoacyl synthase (KS) domains in PKS sequences from *Aspergillus* genomes (Fig. 6). While the size and complexity of full PKS sequences complicates phylogenetic analyses, KS domains exhibit tight clustering patterns and are a useful proxy for exploring the evolutionary relationship of PKSs [70]. To build the network, we first retrieved any sequences in the NCBI protein database from *Aspergillus* species containing hits to the cond_enzymes superfamily (accession: cd00327). This superfamily contains a variety of enzymes catalyzing decarboxylating and non-decarboxylating Claisen-like condensation reactions, covering the spectrum of FASs and PKSs. In total, 2923 sequences were retrieved. Using synthaser, we predicted and classified the domain architectures of each retrieved sequence, then extracted the sequence of each KS domain. This consisted of 95 FAS (25 alpha subunit), 292 hybrid PKS-NRPS, 1991 PKS (35 Type III, 221 PR-PKS, 583 NR-PKS and 960 HR-PKS, with 36 furthest annotation Type I, 156 furthest PKS) and 545 thiolases. This dataset was then extended with the PKS, PKS-NRPS and FAS sequences from the MIBiG database analysed above (137 PKS, 31 PKS-NRPS and 24 FAS).

All versus all sequence comparisons of the extracted KS domains were performed using DIAMOND [45], which were then used to construct a similarity network in CytoScape [71]. Mapping of orthogonal data to sequence similarity networks has been shown to be a powerful approach for revealing themes within biological sequence data [72]. Thus, we mapped domain architecture predictions of the parent PKS sequences generated using synthaser to the KS domain network (depicted in Fig. 6 by colour scheme) to explore their relationships.

Four distinct subnetworks were formed within the KS domain network, corresponding to the four broad classification groups of KS domain-containing sequences. One subnetwork contained mostly highly-reducing and partially-reducing PKS (Fig. 6 top-left), and was clearly separate from, but related to, another subnetwork consisting of hybrid PKS-NRPS sequences (top-right). Non-reducing PKS formed another clear subnetwork (bottom-left), as did domains from fatty acid synthases (bottom-right).

Perhaps the most powerful aspect of the similarity network approach is its ability to reveal outliers; a characteristic we wished to exploit for the purpose of genome mining for biosynthetic novelty. We were immediately drawn to two specific sequence clusters, which were clearly demarcated from the other members of their respective subnetworks thanks to the architecture-mapped colour scheme (circled in Fig. 6). The first cluster falls within the hybrid PKS/NRPS subnetwork and consists of sequences where the NRPS module precedes the PKS module, instead of the typical PKS-NRPS arrangement. Comparatively few NRPS-PKS have been characterised in the literature. The first reported fungal NRPS-PKS was the synthase involved in the biosynthesis of tenuazonic acid in *Magnaporthe oryzae*, TAS1, which has an NRPS module before a PKS module containing only a KS domain [73]. Later, [74] characterised the swainsonine BGC, containing the NRPS-PKS SwnK, in several fungal species. More recently, [75] characterised a NRPS-PKS enzyme, AnATPKS, capable of producing the amino acid derived $\alpha$-pyrone natural products pyrophen and campyrone B in *Aspergillus niger*. While the cluster contains sequences matching the domain architectures of TAS1, SwnK and AnATPKS, it also includes more variation that could be explored in further studies. Perhaps more interesting was the second cluster, which fell within the non-reducing PKS subnetwork and consisted of non-reducing PKSs with ketoreductase (KR) domains at the N-terminal. As previously outlined, a typical NR-PKS sequence starts with a SAT domain and contains a product template (PT) domain and no reductive (DH, ER, KR) domains [10]. The sequences within this cluster match this template almost exactly, with SAT domains being substituted with KR domains, making them very abnormal. While outliers such as this could result from incorrect gene annotation (i.e. through fusion of separate coding regions), given the otherwise textbook NR-PKS domain architectures, proximity of the KR domain to the KS domain, and the number of homologues that were identified, we do not believe this to be the case. One biosynthetic hypothesis might be that the KR domain performs similar reductive processing steps as they do in HR-PKS and PR-PKS. Future work is required to further characterise these synthases; however, the discovery of such sequences highlights the value of synthaser to genome mining pipelines.

## Discussion

In this paper we describe synthaser, a Python-based software package for automatic prediction, classification and visualisation of domain architectures of multi-domain proteins. Synthaser is capable of fully remote searches using the NCBI’s CD-Search tool, which searches query sequences against domain families stored in the conserved domain database (CDD). This is advantageous to other tools which rely on manually curated local profile HMMs for domain searches, as new domains can be added just by referencing the corresponding CDD identifier. Moreover, as the CDD and its sub-databases are continually curated, any improvements to domain profiles will automatically filter through to predictions generated by synthaser.

Synthaser takes a unique approach in that it explicitly searches for specific domain ‘islands’ during domain architecture prediction (Fig. 2). This differs from other tools that select purely for top scoring domains, which can include broader domain families encompassing multiple smaller domains. For instance, a CD-Search of any Type I PKS sequence will contain the domain family PksD, which consists of both KS and AT domains (visible in Fig. 2). While this may be preferable when looking at the overall similarity of two proteins, the goal of synthaser is to instead predict exact domain architectures, reporting every distinct domain found within each sequence. Thus, synthaser can be superior when precise labelling of domains within a sequence is desired.

Another advantage of synthaser is its ability to analyse fungal megasynthases at (pan-)genomic scale. There is currently no tool available that can characterise, classify and display the domain architectures of all PKSs and NRPSs either within a genome or across multiple genomes. As we demonstrate above in our similarity network of KS domains, this can form the basis of a genome mining strategy for uncovering unique synthases encoding potentially novel metabolites.

Domain architecture prediction and classification in synthaser is controlled by an underlying rule file, which can be freely modified by the user. The rule file consists of three components: classes containing CDD domain families which correspond to domain islands, classification rules, and the rule evaluation hierarchy. This allows for a level of flexibility not possible in tools which rely on manually curated profile HMMs. In addition, we provide a web application (https://gamcil.github.io/synthaser) which allows users to easily add, delete or modify domains and classification rules. The default rule file can be loaded for editing by the click of a button, enabling users to tweak it as necessary for their purposes. Moreoever, we can foresee synthaser being completely repurposed, via its rule system, for the analysis of other multi-domain protein families, outside of the scope of secondary metabolites.

In the current paper, we extensively demonstrated the use of this system above in the context of fungal secondary metabolite megasynthases; the corresponding rule file is distributed with, and is the namesake of, the tool. Indeed, synthaser has already seen use in the analysis of fungal biosynthetic gene clusters in our own group [19, 69]. We previously outlined strategies for genome mining for BGCs encoding novel small molecules, or those encoding new or improved bioactivities [22]. Analysis of domain architecture is a key step in uncovering such molecules, as unusual domain architectures could potentially encode unusual chemistry. Such an approach has already been fruitful across several classes of synthase, including PKS, NRPS and terpene synthases [63, 76, 77]. Synthaser makes this analysis significantly more convenient, automating both the prediction and classification stages for sequences in batch, without the need for curation of local domain profiles, or maintenance of local profile databases. In addition, synthaser provides the genbank module, which is capable of directly parsing antiSMASH-generated GenBank format files for megasynthase sequences. If local analysis is desired, synthaser does possess the ability to both download profile databases from the NCBI using its getdb module, as well as perform local searches using RPS-BLAST (provided it it is installed on the system).

Synthaser generates comprehensive visual and text result outputs. The visualisations are fully interactive, allowing for changes to sequence size and shape, as well as other convenient functionalities such as the extraction of domain sequences to FASTA files. The text output reports the length and domain architectures of each query sequence, grouped by their classifications. This can also be generated in tabular formats, such that it can be easily imported into spreadsheet software or incorporated into larger bioinformatic pipelines.

The synthaser approach does have some caveats. While synthaser’s remote search capabilities are its biggest advantage, this also means that an internet connection is required to use the tool. Moreover, certain sequence features indicated by the web CD-Search tool, such as the active sites of certain domains, are not available in synthaser results. Perhaps the largest drawback is that the specificity of domain predictions is limited by the domain profiles within the CDD. This has a couple of consequences. Firstly, distinct but functionally related domains generally cannot be separated during a search. For example, acyl carrier protein (ACP) domains in FAS and PKS and peptidyl carrier protein (PCP)/thiolation (T) domains in NRPS, which are structurally and functionally related, hit the same CDD profiles in a CD-Search run. Synthaser attempts to alleviate this issue by allowing domains to be renamed based on the classification of the protein; in the previous example, synthaser will keep the ACP name within a PKS or FAS, but change it to a T (thiolation) if found in a NRPS. Secondly, certain domains may fail to be detected if the corresponding domain profiles are weakly defined. In these scenarios, synthaser will also fail to report the missing domains. However, this is made very clear in the synthaser visual output, as large gaps in sequence can be seen where missing domains should be (e.g. the NR-PKS N-terminal SAT domain, as shown in Additional file 1: Fig. S2), hopefully prompting further investigation. As curation of the CDD continues, and the quality of domain profiles improves, so to will the predictions given by synthaser.

In summary, synthaser is a powerful tool for the characterisation and classification of multi-domain protein architecture. Synthaser offers both local and remote search capabilities, which utilise the curated domain profiles in the NCBI’s conserved domain database. Its intuitive visualisations, as well as text summaries, allow interesting domain architectures to become immediately obvious. While synthaser is distributed with the fungal megasynthase rule set detailed in this paper, the flexibility of the rule system, as well as the easy to use rule generator web application, means synthaser could readily be repurposed for the study of any multi-domain protein family. Thus, synthaser is a valuable addition to not only the natural products genome mining toolbox, but potentially to any area where multidomain proteins are of interest.

## Supplementary Information

**Additional file 1**: **Fig. S1.** Schema of the synthaser JSON rule file.** Fig. S2**. Synthaser visualisation of domain architectures of non-reducing polyketide synthase (NR-PKS)sequences involved in meroterpenoid biosynthesis showing gaps in N-terminal regions of BAV69313.1 andEAU29529.1.** Fig. S3**. Extract from multiple sequence alignment of N-terminal regions of non-reducing polyketidesynthase (NR-PKS) sequences involved in meroterpenoid biosynthesis showing conservation of the starterunit:ACP transacylase (SAT) domain active site GXCXG motif.

**Additional file 2**: **Table S1. ** MIBiG sequences.** Table S2**. NCBI network.

## Data Availability

Synthaser is freely available from GitHub (https://github.com/gamcil/synthaser) and PyPI (https://pypi.org/project/synthaser) under a MIT license. The datasets and scripts generated and analysed during the current study are available in a GitHub repository (https://github.com/gamcil/synthaser_scripts).
