# Supplementary material for: Synthaser: a CD-Search enabled Python toolkit for analysing domain architecture of fungal secondary metabolite megasynth(et)ases
Source: Fungal Biol Biotechnol. 2021 Nov 11;8:13. doi: 10.1186/s40694-021-00120-9 (PMC8582187; doi:10.1186/s40694-021-00120-9)
Supplement: Supplementary file 1 — Additional file 1: Fig. S1. Schema of the synthaser JSON rule file. Fig. S2. Synthaser visualisation of domain architectures of non-reducing polyketide synthase (NR-PKS)sequences involved in meroterpenoid biosynthesis showing gaps in N-terminal regions of BAV69313.1 andEAU29529.1. Fig. S3. Extract from multiple sequence alignment of N-terminal regions of non-reducing polyketidesynthase (NR-PKS) sequences involved in meroterpenoid biosynthesis showing conservation of the starterunit:ACP transacylase (SAT) domain active site GXCXG motif. [file 40694_2021_120_MOESM1_ESM.pdf]

synthaser: a CD-Search enabled Python toolkit for analysing domain architecture of fungal secondary metabolite megasynth(et)ases

Cameron L.M. Gilchrist*[1], Yit-Heng Chooi*[1]

[1]School of Molecular Sciences, The University of Western Australia, 35 Stirling Hwy, Crawley, 6009

E-mail: cameron.gilchrist@research.uwa.edu.au, yitheng.chooi@uwa.edu.au

List of Figures

S1 Schema of the synthaser JSON rule file. 2

S2 synthaser visualisation of domain architectures of non-reducing polyketide synthase (NR-PKS) sequences involved in meroterpenoid biosynthesis showing gaps in N-terminal regions of BAV69313.1 and EAU29529.1. 3

S3 Extract from multiple sequence alignment of N-terminal regions of non-reducing polyketide synthase (NR-PKS) sequences involved in meroterpenoid biosynthesis showing conservation of the starter unit:ACP transacylase (SAT) domain active site GXCXG motif. 4

```
{
  "domains": [
    "name": <string: name of the domain island>,
    "domains": [
       {
        "pssm":        <integer: PSSM number>,
        "accession":   <string: accession>,
        "name":        <string: family name>,
        "length":      <integer: PSSM length>,
        "bitscore":    <float: threshold bitscore>,
        "superfamily": <string: superfamily>,
       }
    ]
  ],
  "rules": [
    {
      "name":     <string: name of classification rule>,
      "domains": [<string: domain island name>],
      "filters": [
        {
          "type":     <string: domain island name>,
          "domains": [<string: domain family accession to select>]
        }
      ],
      "renames": [
        {
          "from":    <string: domain island being renamed>,
          "before": [<string: islands that must occur after renaming island>],
          "after":  [<string: islands that must occur before renaming island>],
          "to":      <string: new domain island name>
        },
      ],
      "evaluator": <string: rule evaluation string>,
    }
  ],
  "hierarchy": [
    {
      "title":     <string: name of classification rule>,
      "children": [
       {
         "title": <string: name of child classification rule>,
         "children": [ ... ]
       }
      ]
   }
  ]
}
```

Figure S1: Schema of the `synthaser` JSON rule file. Fields used by the rule generator web application (unique identifiers, labels for HTML elements, etc) but not `synthaser` itself have been ommitted for clarity. A more complete example is the fungal megasynthase rule set, which is distributed alongside the source code at `https://github.com/gamcil/synthaser` (`rules.json`).

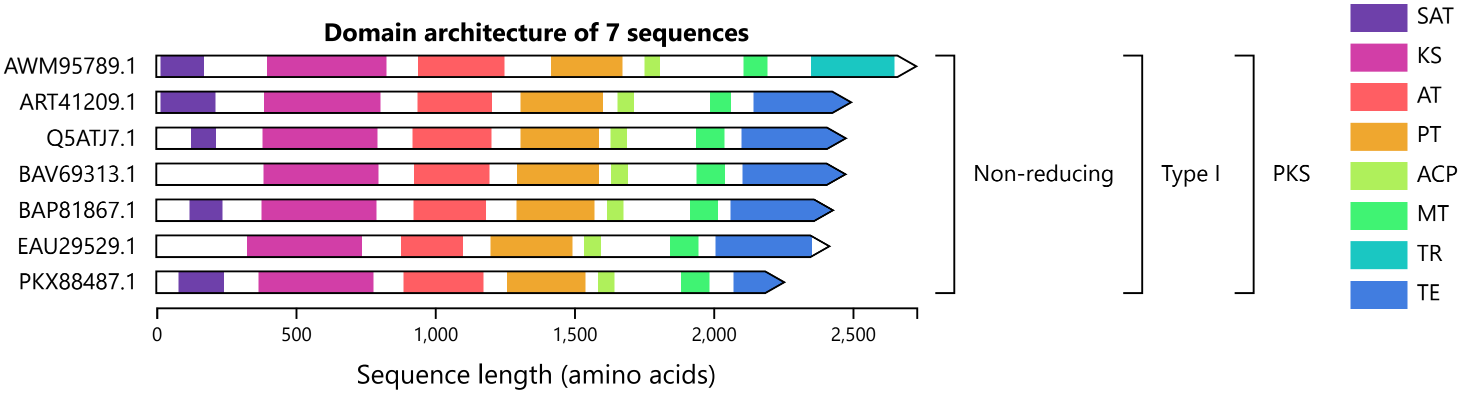

Figure S2: `synthaser` visualisation of domain architectures of non-reducing polyketide synthase (NR-PKS) sequences involved in meroterpenoid biosynthesis showing gaps in N-terminal regions of BAV69313.1 and EAU29529.1.

```
               120       130       140       150       160       170       180
                 |         |         |         |         |         |         |
 AWM95789.1    RQSPTGLA--EGKEAIGFCTGILSAF-AVASSHDVCDLAKYGAAAMRLGMLVGLVVDCED
   Q5ATJ7.1    ELKEDK-N-YDVCDIQGFCVGFLAAIAAACWSDNEDEFGKVVSTVLRLAVYIGAAVDLDE
 EAU29529.1    ------------KKSPGFCVGYLAAV-AACWETDQTEFPKAVATMLRIAVCIGAVVDLDE
 BAV69313.1    EFKEAGVN-CDIKSMQGFCAGYLAAV-AACWEKDQSEFSKVVATMVRTAIFIGAAVDLDE
 ART41209.1    KFKEESQNRCRIVDAQGFCVGFLAAV-AVASSNDSNEFEDIASTMIRLAVCIGAAVDLDG
 BAP81867.1    RLRKRGSS-LQIKDVQGFCVGFLAAT-AVASAHDETQFRSIVAKVIRLAVCVGALVDLNE
 PKX88487.1    KVKETMKG-FQARDVQGFCVGFLAAT-AVAASCDETAFRALVSKIIRLAVCIGGLVDLDE
```

Figure S3: Extract from multiple sequence alignment of N-terminal regions of non-reducing polyketide synthase (NR-PKS) sequences involved in meroterpenoid biosynthesis showing conservation of the starter unit:ACP transacylase (SAT) domain active site GXCXG motif.
